# Future directions for medicinal chemistry in the field of oligonucleotide therapeutics

**DOI:** 10.1261/rna.079511.122

**Published:** 2023-04

**Authors:** Jonathan Hall

**Affiliations:** Institute of Pharmaceutical Sciences, Department of Chemistry and Applied Biosciences, ETH Zurich, 8093 Zurich, Switzerland

## Abstract

In the last decade, the field of oligonucleotide therapeutics has matured, with the regulatory approval of several single-stranded and double-stranded RNA drugs. In this Perspective, I discuss enabling developments and likely future directions in the field from the perspective of oligonucleotide chemistry.

## INTRODUCTION

Most researchers in the pharmaceutical industry in the early 1990s believed that oligonucleotides stood little chance of success as a new class of drug. Textbook knowledge stated that large polyanionic structures do not enter cells (Gennis 1989) and thus, oligonucleotide drugs should not function in vivo. Furthermore, a manufacturing process for oligonucleotides seemed improbable, and therefore, there was no credible business case for such a class of therapeutics. In spite of this, today 13 oligonucleotide drugs have been approved by regulatory authorities (comprehensively reviewed in reference, Crooke et al. 2021). It is perhaps time to rewrite some chapters of the textbooks.

Today, oligonucleotide drugs are approved for use as medicines in the liver, the central nervous system (CNS), the skeletal muscle and the eye, and there are good reasons to believe that they will soon be validated in the lung, the kidney, and the bone marrow. Thus, oligonucleotides are now established as a major class of therapeutics, behind small-molecule drugs and therapeutic proteins.

At the annual Oligonucleotide Therapeutics Society meeting in Phoenix, I was asked by a young chemist where I thought that medicinal chemistry could play a role in the future of oligonucleotide drugs.

### Medicinal chemistry milestones in oligonucleotide therapeutics

Looking back over 30 yr, a handful of milestones in the chemistry of oligonucleotides stand out. Oligonucleotide drugs are large, chemically synthesized structures, and therefore optimization of their pharmacodynamics (PD) and pharmacokinetics (PK) properties fell under the responsibility of medicinal chemists. Pioneering work was carried out by chemists through the 1980s and the 1990s, during which the ribonucleotide structure was systematically modified in efforts: (i) to protect single-stranded antisense oligonucleotides (ASOs) against metabolic degradation, while retaining their ability to hybridize with their targets and to recruit cellular effector enzymes; and (ii) to remain accessible via solid-phase synthesis. The experience gained in these areas streamlined efforts a decade later with a second emerging class of oligonucleotide drugs, the double-stranded small interfering RNAs (siRNAs) (Elbashir et al. 2001). In parallel with this work, major advances were made with oligonucleotide synthesizers, both in terms of synthesis throughput and synthesis scale. The introduction of 96-well machines, such as the Mermade 192, allowed researchers to synthesize oligonucleotides in “high-throughput.” This meant that instead of struggling to predict possible binding sites for potent oligonucleotides on a target mRNA with the help of RNA folding programs, or by assessing GC-content, it became routine in industry to synthesize and screen hundreds of reagents in a brute-force approach to identify experimentally and unambiguously the “best” oligonucleotide. In turn, access to large screening datasets powered the use of machine learning methods that revealed some of the sequence-dependent properties of potent oligonucleotides, as described in 2005 with siRNAs (Huesken et al. 2005). Meanwhile, at the opposite end of the synthesis spectrum, large capacity synthesizers were introduced, providing gram quantities of oligonucleotide reagents for routine testing in animal disease models, including nonhuman primates. Today, the OligoProcess synthesizer produces up to 15 kg of oligonucleotide in single batches. With these developments ongoing, the field had momentum.

### Ribose modifications in single-stranded RNA drugs

**FIGURE 1.**
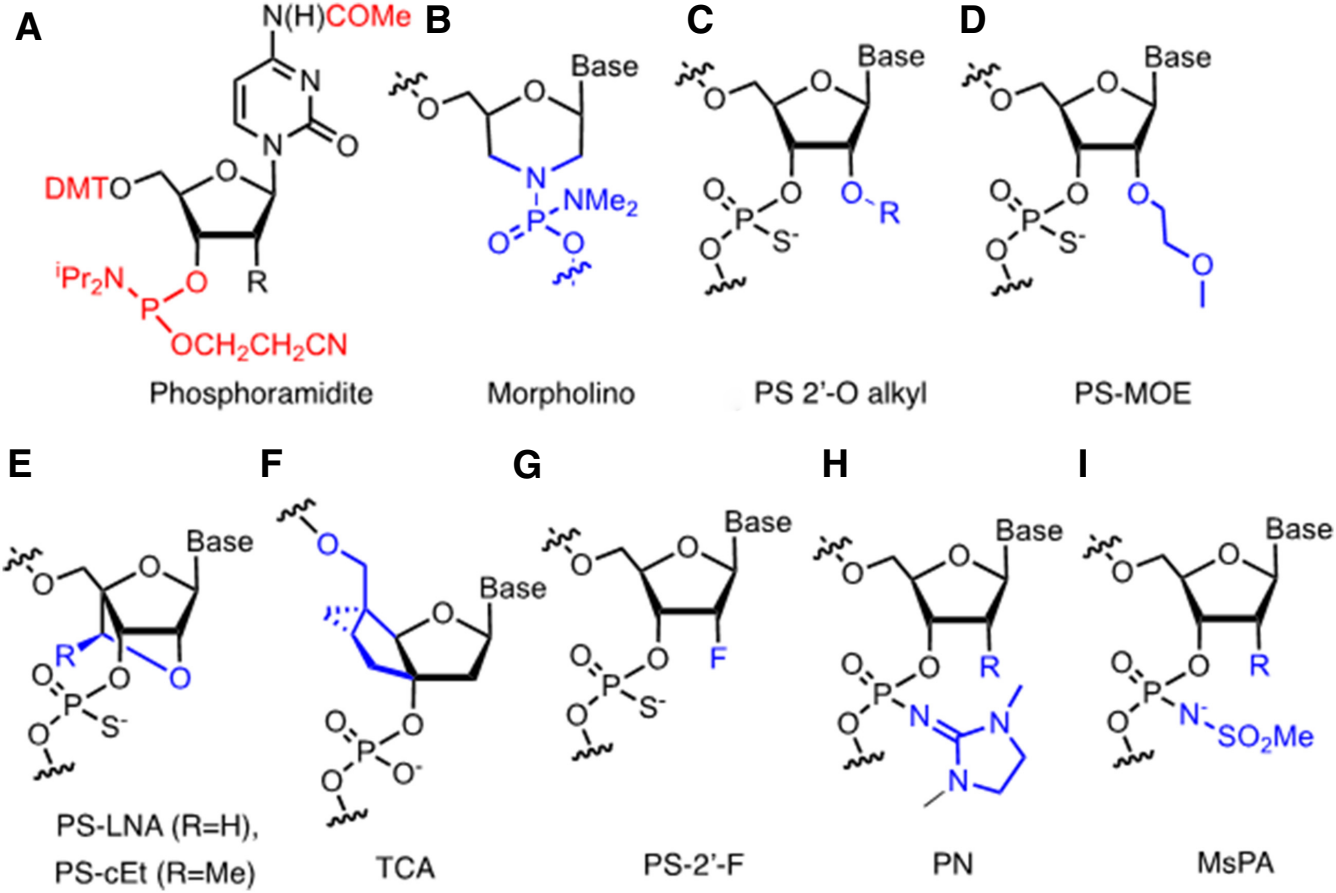
Ribonucleoside modifications present in advanced oligonucleotide drug candidates. (*A*) Conventional cytidine phosphoramidite building block that is activated by tetrazole for coupling to the growing oligonucleotide on solid support; the reactive P (III) group and protecting groups in red. (*B*) Morpholino group. (*C*) Modifications on the 2′-O position of the ribose groups. (*D*) MOE group. (*E*) LNA and cEt groups. (*F*) Tricyclo DNA group. (*G*) 2′-F groups. (*H*) Phosphoryl guanidine (PN) group. (*I*) Mesyl phosphoramidite (MsPA) group. Modifications (C–*E*) are usually PS-modified in oligonucleotide drugs; 2′-F groups are either PS or PO-modified in siRNAs; groups (*B*–*E*,*G*–*I*) contain chiral internucleotide linkages. Blue illustrates the modification compared to native DNA and RNA structure.

The phosphodiesters of a native DNA or RNA oligonucleotide are quickly degraded by ubiquitous nucleases in vivo. Hence, medicinal chemists were tasked with modifying oligonucleotide structures to render them resistant to metabolism. However, researchers were alarmed to find that even minor modifications to the ribonucleotide unit of an ASO could severely reduce its affinity for a complementary RNA. Hence, over a period of two decades, hundreds of nucleoside modifications were designed, synthesized and tested in academia and industry, in search of the “perfect” modification (Wan and Seth 2016). The synthetic chemistry was resource-intensive, monotonous, and demanding. In most cases, it necessitated the synthesis of the four nucleosides as stable but reactive phosphoramidites (Fig. 1A), with protecting groups on the exocyclic amino groups of the nucleobases, and good solubility in acetonitrile solvent. These building blocks were subjected to solid phase synthesis, then harsh ammonia treatment, followed by purification and characterization. The resultant oligonucleotide was then evaluated for its binding affinity and selectivity toward a complementary RNA in in vitro assays. Not surprisingly, the rate of attrition was high and most of these modifications fell by the wayside; very few reached clinical evaluation and drug approval.

Among the successful modifications, one of the most unusual was the phosphorodiamidate morpholino oligonucleotide (PMO) (Fig. 1B). Its elegant synthesis involves oxidative-mediated ring opening of the ribonucleoside, followed by ring closure with reductive amination, to produce a nucleobase-substituted morpholine cycle. The morpholines are linked by a phosphorodiamidate backbone (Iversen 2001). This chemistry was tested in the clinic with the splice-switching oligonucleotide eteplirsen. The target of eteplirsen is the pre-mRNA of dystrophin in skeletal muscle cells, to which it binds and alters splicing so as to exclude a deleterious exon. The approval of eteplirsen (2016) for the treatment of Duchenne muscular dystrophy (DMD) was controversial, due to the low level of correction that the drug reportedly achieves in the skeletal muscles of patients (Kesselheim and Avorn 2016). Nevertheless, its approval paved the way for three subsequent PMO drugs (golodirsen, vitolarsen; Aartsma-Rus and Corey 2020, casimersen) to address other disease-causing mutations in two other exons of dystrophin for DMD treatment (Crooke et al. 2021). The morpholino drugs were notable as one of the earliest demonstrations that an antisense drug could rescue a genetically derived, loss-of-function phenotype by altering the splicing of an mRNA.

Without doubt, the most successful means to modify DNA and RNA for therapeutic applications comprised two concomitant changes to the structure: exchange of the phosphodiester (PO) for the phosphorothioate (PS) group, as well as substitution of the ribose 2′-*O*-position (Fig. 1C; Manoharan 1999; Wan and Seth 2016). The pioneering work of F. Eckstein had shown that incorporation of PS linkages into the backbone of an oligonucleotide greatly improves its hydrophobicity and nuclease stability (Eckstein 2014). Fortunately, the PS group was easily adapted to solid-phase synthesis protocols and the modification was found—unexpectedly—to facilitate entry of PS oligonucleotides into cells (Loke et al. 1989; Beltinger et al. 1995; Stein et al. 2010; Miller et al. 2016). Furthermore, PS linkages in an ASO result in its weak binding to serum proteins, such as human albumin that retards its renal clearance (Gaus et al. 2019) and permits a wide distribution of a drug in vivo (Geary et al. 2015).

Substitution of the hydroxyl group at the 2′-position of the ribose was an obvious avenue of investigation for chemists (Manoharan 1999). A variety of different substituents were studied, ranging from small alkyl groups to alkyl chains containing aromatic, halogenated and amino groups. The most significant breakthrough came with the introduction of the 2′-*O*-methoxyethyl (MOE) group, described in a 1995 Helvetic publication by P. Martin (Fig. 1D; Martin 1995). The MOE group imposes a C3′-*endo* conformation on the riboses of an oligonucleotide, which enhances hybridization affinity and selectivity for target RNAs (Teplova et al. 1999). Furthermore, in combination with the PS linkage, an MOE substituent renders an oligonucleotide highly stable to *endo*- and *exo*-nucleases.

The MOE modification is today the most widely used chemical modification of single-stranded oligonucleotide drugs (for review, see Hall and Hill 2023). The modification was clinically validated with the approval of mipomersen, a 20-mer “gapmer” PS oligonucleotide bearing five MOE-modified riboses flanking a 10-mer DNA “window.” The DNA segment recruits RNase H1 to the target mRNA, thereby mediating its cleavage and terminating synthesis of the target protein (Monia et al. 1993). Mipomersen targets the liver as a treatment for familial hypercholesterolaemia (FH), a rare disorder of low-density lipoprotein cholesterol (LDL-C) metabolism (Raal et al. 2010). Despite mipomersen not being a commercial success, it generated spectacular data and was celebrated by the field as the first of the new-generation oligonucleotide drugs, able to suppress selectively the expression of a deleterious protein (Kastelein et al. 2006; Parham and Goldberg 2019).

The approval of mipomersen in the USA (2013) was quickly followed by that of nusinersen (2016), a breakthrough treatment for spinal muscular atrophy (SMA). Nusinersen is a fully PS-MOE-modified, 18-mer ASO that binds to *SMN2* pre-mRNA and alters its splicing, to switch on production of a functional SMN protein (Hua et al. 2007; Corey 2017). It was the first oligonucleotide drug to work in the nervous system, confirming findings in the late 1990s that intrathecal delivery into the cerebral spinal fluid was a viable means to administer MOE oligonucleotides into the CNS (Barclay et al. 2002; Dogrul et al. 2002). Also, it is the only oligonucleotide to date to achieve “blockbuster drug” status.

A number of alternative ribose modifications for single-stranded RNA drugs are also worthy of mention. They include the structurally complex bicyclic “locked” nucleic acid (LNA, cEt) modifications (Koshkin et al. 1998; Seth et al. 2008) and tricyclic deoxyribose (TCA) derivatives (Renneberg and Leumann 2002) that endow oligonucleotides with very high RNA-binding affinities (Fig. 1E,F). However, for a variety of reasons, they have either fallen at (e.g., miravirsen; Janssen et al. 2013), or not yet cleared (e.g., danvatirsen; Hong et al. 2015), the last hurdles before regulatory approval. Intuitively, it seems likely that some of these structures will eventually achieve success in the clinic.

### SiRNAs and oligonucleotide conjugates

The gapmer design of ASOs provided a workable solution for chemists aiming for a compromise between stability, affinity, and RNase H-compatibility. For siRNAs, the main difficulty with the PD properties was to achieve nuclease stability of the double-stranded RNA (dsRNA) in view of the sensitivity of the RNAi mechanism to structural modifications in the two strands (passenger and guide) (Bramsen et al. 2009). Furthermore, the mainstay substituents of antisense oligonucleotides, such as MOE, are poorly accepted by the RISC (RNA-induced silencing complex) machinery in many (but not all; Dorn et al. 2004) positions of the siRNA duplex. Eventually researchers from siRNA Therapeutics and Alnylam Pharmaceuticals converged on the replacement of all ribonucleotides in an siRNA with intricate arrangements of 2′-*O*-methyl (OMe) and 2′-fluoro (F) nucleotides (Fig. 1G; Foster et al. 2018). These fully modified siRNAs are then capped with a few terminal PS groups to top-up nuclease stability. This structural format was not effective in in vivo applications, since in contrast to single-stranded oligonucleotides, dsRNAs do not bind serum proteins and are quickly excreted from the body (Biscans et al. 2018). Furthermore, they do not undergo gymnosis—unaided uptake into cells—in contrast to their single-stranded counterparts (Dowdy 2017). This hurdle was countered by their formulation with multicomponent lipid nanoparticles (LNPs) (Bost et al. 2021), which were used for the first siRNA drug patisiran in the treatment of hereditary transthyretin-mediated amyloidosis (Adams et al. 2018). However, LNPs have mostly fallen out of favor for siRNA formulations, because of the complexity of their composition (Akinc et al. 2008; Jayaraman et al. 2012; Dowdy 2017) and their perceived potential for long term toxicity. Instead, the RNA field adopted a different strategy for siRNA delivery in vivo—the oligonucleotide conjugate.

The idea of conjugating functional groups to oligonucleotides to improve their PD and PK properties is decades old (for an excellent early review see, Manoharan 2002). A variety of innovative functional groups have been explored by chemists, ranging from intercalators (Asseline et al. 1984), peptides (Turner et al. 2005), stable metal complexes (Hall et al. 1996), and polyamines (Menzi et al. 2017). Today, for improved siRNA (and ASO) delivery, oligonucleotide conjugates can be grouped into those that improve systemic circulation, those that aid cellular uptake, or those that do both (Juliano 2016).

Hydrophobic groups such as cholesterol and other lipids were seen to alter the distribution of siRNAs from liver (Soutschek et al. 2004; Osborn et al. 2018), producing measurable target suppression in kidney, heart, lung, fat, and muscle (Biscans et al. 2018). Higher hydrophobicity of the conjugate group led to greater tissue retention of the siRNA, although higher siRNA accumulation in a tissue does not correlate with higher gene silencing in cells of the tissue, as reported by several groups (Halloy et al. 2020).

**FIGURE 2.**
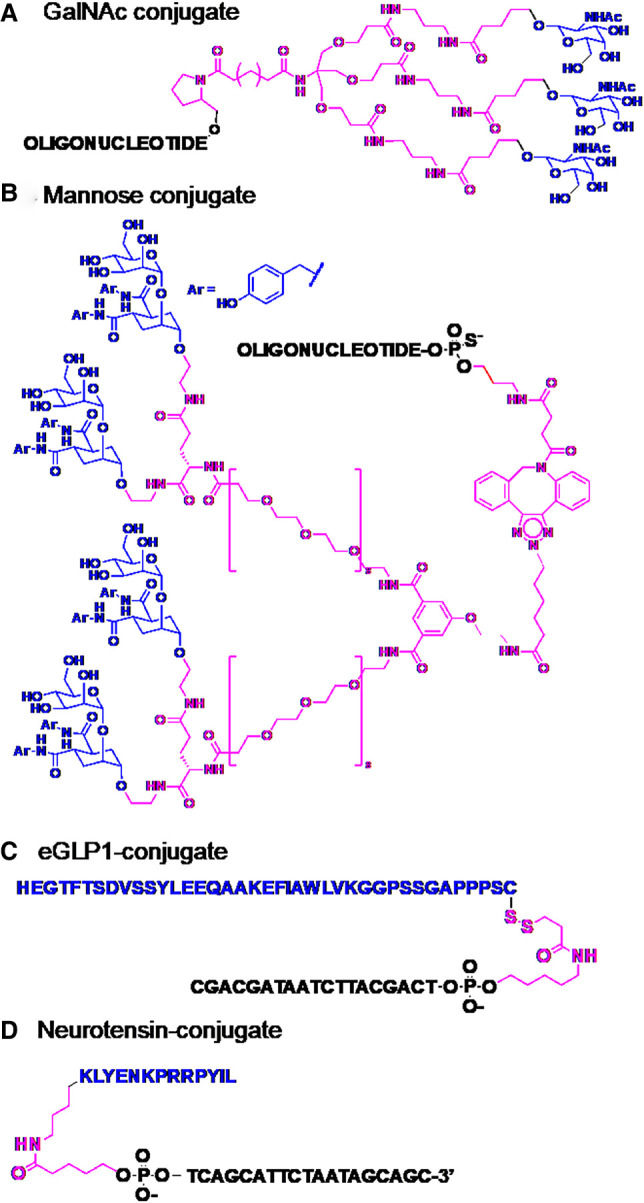
Oligonucleotide conjugates. (*A*) The GalNAc conjugate used for hepatic delivery of ASOs and siRNAs (Nair et al. 2014). (*B*) Mannose ligands used to deliver siRNAs to macrophages and dendritic cells via the CD-206 receptor (Uehara et al. 2022). (*C*) Peptide conjugate used to deliver Malat1-targeting gapmer oligonucleotides to pancreatic beta-cells (Knerr et al. 2021). (*D*) Neurotensin peptide used to deliver Malat1-targeting gapmer oligonucleotides to various regions of the brain (Nikan et al. 2020). Targeting ligands shown in blue; linker groups shown in pink.

The conjugation of receptor-targeting ligands to ASOs and siRNAs has been championed by researchers at Ionis Therapeutics (Prakash et al. 2014) and Alnylam Pharmaceuticals (Nair et al. 2014). The conjugation of a targeting ligand composed of three *N*-acetylgalactosamine (GalNAc) moieties to the 3′-end of the siRNA passenger strand stands alone as a breakthrough for the RNAi field (Fig. 2A; Nair et al. 2014). GalNAc ligands show high affinity for asialoglycoprotein receptors (ASGPRs), expressed in hepatic cells. Upon ligand binding, ASGPRs undergo endocytosis, transporting the conjugated siRNA into cells (Spiess 1990). There, the conjugate is metabolically cleaved releasing its siRNA into the cytosol. The GalNAc group is so effective for hepatocyte delivery, that drug potency is improved by 10- to 30-fold, compared to nonconjugated ASOs (Crooke et al. 2019; Debacker et al. 2020). This conjugation strategy was clinically validated for siRNAs with the approval of givosiran targeting delta-aminolevulinic acid synthase 1 (*ALAS1*), for treating the rare metabolic disorder acute hepatic porphyria, as well as inclisiran, the first RNA drug to treat a common disease—atherosclerotic cardiovascular disease (Santulli et al. 2021).

The consequences of GalNAc-targeting for the oligonucleotide therapeutics field cannot be over-stated, and leading siRNA and antisense companies have stacked their clinical pipelines with GalNAc conjugates (Debacker et al. 2020). Researchers are now racing to identify the “next GalNAc” (Dowdy 2017). On paper, the approach appears daunting (Juliano 2016). For a given target cell type, one needs to identify a highly expressed surface receptor that is internalized upon ligand binding, for which a ligand is available and can be attached at an appropriate position of the oligonucleotide or the siRNA. In addition, the site of conjugation and the composition of the linker should not attenuate the ability of the ligand to interact with its receptor or prevent the receptor from internalizing. Three main classes of conjugate ligands have been investigated for targeting through specific receptors: carbohydrates, peptides, and antibodies.

Following the GalNAc example, a tetra-valent mannose ligand was conjugated to siRNAs for selective delivery to CD206-expressing macrophages and dendritic cells in vitro and in vivo (Fig. 2B; Uehara et al. 2022). Similar to the ASGPR, the CD-206 receptor is expressed selectively on the cell surface and undergoes fast recycling. Also, as for the GalNAc group, the multivalent ligand showed superior potency over a monovalent ligand, demanding the design and synthesis of a long, structurally complex linker group (Fig. 2B). In mice, these conjugates accumulated and elicited gene silencing in CD-206-expressing cells.

The most advanced example of receptor mediated targeting is that of the Glucagon-Like-Peptide-1 agonist (GLP-1), which was developed to target specifically pancreatic beta cells, where GLP1R expression is restricted (Ämmälä et al. 2018). Gapmer ASOs conjugated to the 37-amino acid peptide GLP-1 inhibited their targets in the pancreatic cells (Fig. 2C). Astonishingly, these ASOs are devoid of effects at low doses in liver, after systemic administration to *ob/ob* mice. An analogous approach is being pursued for targets in the brain, using ASOs or siRNA conjugated to a short 13-amino acid neurotensin peptide that binds with high affinity at the sortilin receptor (Fig. 2D; Nikan et al. 2020). The neuropeptide was conjugated to morpholino oligonucleotides. However, the reagents have exhibited relatively modest improvements in splice-modulating activity in the cortex and striatum of mice after intracerebroventricular injection.

New ways to use oligonucleotide conjugation as a means to improve drug trafficking are underway, as our understanding of how oligonucleotides trafficking in cells and in vivo increases (Juliano 2016). For example, ancillary groups that aid endosomal escape of the oligonucleotides in cells or that help traffic an oligonucleotide to the nucleus would be of potentially high value (Dowdy 2017). Such initiatives are supported by the development of new highly sensitive hybridization-based analytical techniques that can quantify oligonucleotides in individual protein complexes (Brunschweiger et al. 2016) or compartments of the cell (Godinho et al. 2017) or in distinct tissues/organs of the body (Boos et al. 2013).

### Phosphorothioate linkages—the Dr. Jekyll and Mr. Hyde of oligonucleotide therapeutics

The PS-linkage is an indispensable part of many oligonucleotide drugs and is likely to remain so for the foreseeable future. In the early phases of the field, it powered advances in the technology, thanks to its favorable PK properties, its metabolic stability, its ease of synthesis and its compatibility with the RNase H mechanism. However, the PS-linkage is often maligned for its toxicity (Shen et al. 2019; Crooke et al. 2021), its metabolic instability in some sequence contexts and the hidden secrets of its isomeric composition. For some applications in vivo, efforts have been made to reduce the number of PS groups in an oligonucleotide, for example, by substituting selected linkages with stable PO-groups (Wahlestedt et al. 2000; Barclay et al. 2002), with alkyl phosphonates (Migawa et al. 2019), with phosphoryl guanidine (PN) groups (Fig. 1H; Kandasamy et al. 2022a,b) or with mesyl phosphoramidite (MsPA) groups (Fig. 1I; Miroshnichenko et al. 2019; Hammond et al. 2021). The PN and MsPA groups represent relatively new chemistries that are highly resistant to nucleases and are easily incorporated into the solid-phase synthesis cycle by substituting an azide synthon for sulfur during P (III) to P (V) conversion (Zhukov et al. 2021).

**FIGURE 3.**
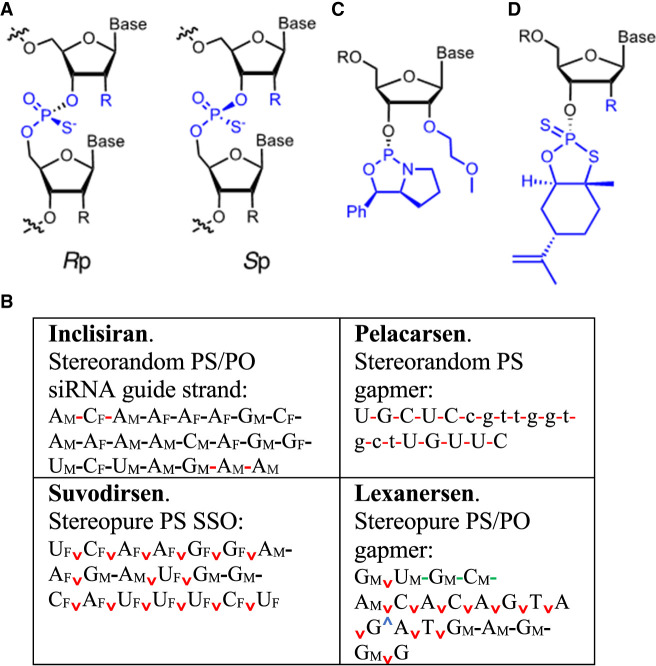
Stereopure synthesis of PS-oligonucleotides. (*A*) The *R*p and *S*p diastereoisomers of a PS linkage. (*B*) Selected stereorandom and stereopure oligonucleotides tested in humans (5′–3′). (*C*) Phosphoramidite building block used to synthesize stereopure PS oligonucleotides (R is H, alkyl or silyl groups). (*D*) P (V) building blocks used to synthesize stereopure oligonucleotides. (-: *R*p/*S*p mixed PS linkage; -: PO linkage; v: *S*p PS linkage; ^: *R*p PS linkage; N_M_: 2′-OMe; N_F_: 2′-F; n: deoxyribonucleotide).

During conventional solid phase PS-oligonucleotide synthesis, the coupling of phosphoramidite building blocks (Fig. 1A) occurs with epimerization, mediated by nucleophilic tetrazole activators. Thus, PS stereochemistry is not controlled, and therefore each linkage in the oligonucleotide exists as an approximate 1:1 ratio of *R*p and *S*p diastereoisomers (Fig. 3A). Thus, the siRNA inclisiran with six PS groups comprises up to 64 (2^6^) isomers, whereas the 20-mer ASO pelacarsen (Tsimikas et al. 2020) has 524,288 (2^19^) possible diastereoisomers (Fig. 3B). Ravikumar and Cole studied the influence of various parameters on the *R*p/*S*p ratios produced during the coupling of conventional MOE phosphoramidites (Fig. 1A), including synthesis scale, solid supports, machines, reagent concentrations, tetrazole activators, and phosphodiester protecting groups. They concluded that activators and the phosphate protecting groups had the greatest influence during solid phase synthesis (Ravikumar and Cole 2003). These findings were consistent with later work by T. Wada on RNAs (Oka and Wada 2011) and on (si)RNAs by Jahns et al. (2015) who demonstrated that subtle changes in the *R*p/*S*p composition of PS RNAs in siRNAs significantly affects their properties in cells.

The pharmacological properties of an oligonucleotide are the sum activity of its component isomers. Each diastereoisomer exhibits its own distinct PD and PK properties (Jahns et al. 2015, 2021). In modern conventional drug development, the use of diastereosiomeric mixtures of drugs is avoided wherever possible. However, due to the strict requirement for quantitative coupling reactions during oligonucleotide synthesis, the field of oligonucleotide therapeutics has been exempt from this condition. Recent developments, however, suggest this aspect should be reexamined, since: (a) it is now possible (though challenging) to synthesize antisense PS-oligonucleotides stereospecifically (Iwamoto et al. 2017); (b) a loss of stereochemical reproducibility during manufacturing may have contributed to the failure of the first generation antisense drug mongersen (Arrico et al. 2022); and (c) innovative new P (III) (Fig. 3C; Li et al. 2017) and P (V) chemistry (Fig. 3D; Stec et al. 1991; Knouse et al. 2018) has stirred chemists to revisit methods of oligonucleotide synthesis.

The main premise of stereopure PS-oligonucleotides—besides the obvious benefits of working with a single molecular entity—is that one may be able to influence (improve) distinct PD and PK properties via control of PS stereochemistry, if methods are available to test/synthesize all possible diastereoisomers. For example, it has been demonstrated that some PS diastereoisomers in a stereorandom population of a PS gapmer show exaggerated toxicity, from the chiral interaction of selected PS groups with proteins (Hyjek-Składanowska et al. 2020). This toxicity was attenuated by a switch in the stereochemistry at specific PS centers. Moreover, there is the tantalizing prospect that through interactions with certain proteins, a distinct PS stereochemistry may for example, improve potency, aid target cell uptake or mediate allele-specific targeting etc. Indeed, it is long-known that a DNA segment composed of *R*p centers in a PS gapmer oligonucleotide is RNase H-compatible, but is quickly degraded by nucleases; whereas the *S*p diastereoisomers have better stability but show poor RNase H-compatibility (Koziolkiewicz et al. 1995).

The major breakthrough in the chemistry of stereopure PS-oligonucleotides was the introduction by the Wada group of new P (III) nucleoside building blocks and activators that enable stereospecific coupling on solid phase (Oka and Wada 2011; Nukaga et al. 2012). Initially, coupling yields with this chemistry were not sufficient to produce 20-mer oligonucleotides. However, by tinkering with the substituents on the chiral ancillary and the reaction conditions, chemists from Wave Life Sciences prevailed with the first chemical synthesis of “full-length” stereopure PS oligonucleotides (Iwamoto et al. 2017).

A second outcome of this seminal work was the discovery that a trivalent stereo-motif 3′-*S*p*S*p*R*p-5′ in the DNA segment of a stereopure PS gapmer provides both nuclease stability and RNase H compatibility (Iwamoto et al. 2017), circumventing the longstanding challenge (Wan et al. 2014) of how to exploit stereopurity in the DNA window of a gapmer. The authors demonstrated that the motif functions in gapmer oligonucleotides with different chemistries in the wings, although its benefit was not observed in some oligonucleotides with stereorandom PS-wings (Østergaard et al. 2020).

To date, the properties and applications of fully stereopure PS oligonucleotides have been described in a handful of prominent papers (Iwamoto et al. 2017; Li et al. 2017; Byrne et al. 2021; Liu et al. 2021; Kandasamy et al. 2022a). Critical analysis of the data in these works confirms that a stereodefined PS-backbone shows superior potency and duration of action to its stereorandom counterpart, in vitro and in vivo. Nevertheless, it cannot be forgotten that many factors other than potency play roles on the road to regulatory approval.

Pleasingly, the phosphoryl guanidine and mesyl phosphoramidate groups (Fig. 1H,I) can also be combined with Wada phosphoramidites to yield stereopure amidate linkages (Anderson et al. 2021; Kandasamy et al. 2022a), thereby reducing the PS content of an oligonucleotide without compromising either metabolic stability or stereopurity. The uncharged stereopure PN modification was incorporated into the wings of gapmer oligonucleotides, as well as splice switching oligonucleotides, that showed superior activity to their PS counterparts in the CNS (Kandasamy et al. 2022a). The authors suggested that these enhanced effects occurred through improved oligonucleotide delivery. From the first data with this chemistry, it seems likely that the stereopure PN modification has a bright future in the field.

Stereopure PS-gapmer oligonucleotides were “validated” in clinical trials of suvodirsen (a splice-switching oligonucleotide comprising 2′-OMe and 2′-F ribose modifications to treat DMD), as well as rovanersen and lexanersen (for Huntington`s disease). However, all three front-runner drugs failed to progress in these challenging disease indications, possibly for reasons of insufficient target exposure (DMD) or mechanism-related toxicity (Huntington's disease). However, the next wave of stereopure PS-oligonucleotides is already in clinical trials and therefore it seems likely that the approval of the first such drug is only a matter of time.

## OUTLOOK

The examples of medicinal chemistry discussed in this Perspective—chemical modifications, oligonucleotide conjugates, PS stereochemistry—were selected to highlight three areas of future challenges for medicinal chemists in the oligonucleotide therapeutics field.

Arguably, the need for new ribonucleoside modifications in the field has receded in recent times. This is due to the ready accessibility of MOE and LNA chemistries. When combined with “routine” high-throughput synthesis/screening methods, potent oligonucleotides can be produced against any target for which the sequence is known, as originally envisioned by Zamecnik and Stephenson (Stephenson and Zamecnik 1978; Zamecnik and Stephenson 1978). Furthermore, barriers of intellectual property related to these ribose chemistries have mostly ebbed away, leaving freedom to operate in the field. Reassured by the success of nusinersen that the technology can deliver, many large pharma companies have initiated oligonucleotide (antisense or siRNA) programs. For example, dozens of MOE-oligonucleotides are at various stages of clinical testing (Crooke et al. 2021), sponsored by a variety of companies. Many of these clinical candidates are intended for use in rare diseases, where targets are clinically validated and competition with conventional drug classes is sparse. However, a growing number of programs are directed to the treatment of common diseases, with large patient populations. If only a small fraction of these new programs is clinically successful, it will likely create a strain on contract research organizations for oligonucleotide manufacture. On the other hand, it will also motivate chemists to seek out new methods of oligonucleotide synthesis that are better scalable and “greener” than current methods. Such initiatives may range from the development of new solid supports with higher loadings (similar to peptide solid supports), through solution-phase synthesis (Zhou et al. 2022) to even enzymatic synthesis (Freund et al. 2022).

Recently, many research groups have turned to the area of oligonucleotide conjugates, for enhanced oligonucleotide delivery. Juliano (2016) has described two parts to the delivery problem: first, how to transport the oligonucleotide to the target organ of interest, and then, how to deliver it into the right cellular compartments. Oligonucleotide conjugates offer excellent possibilities to address both objectives, possibly with dedicated conjugate groups for each. However, current oligonucleotide conjugates have high structural complexity for chemical synthesis (see structures drawn in full in Fig. 2). This complicates their development in the areas of synthesis/manufacture, companion analytics, as well as their metabolism and toxicity. These factors can be underestimated by chemists engaged in exploratory research. However, process chemists responsible for preclinical and clinical development of the drugs are sensitive to their large structures, where the conjugated group represents a significant part of the overall structure. Indeed, the manufacturing of inclisiran (Fig. 2A), containing the tri-antennary GalNAc ligand is a formidable achievement. One means to simplify these structures would be to replace carbohydrate- and peptide-targeting ligands with small-molecule ligands that are equally capable of binding selectively and potently to internalizing cell surface receptors. A few reports describe targeting with small-molecule ligands, for example, anisamide (Nakagawa et al. 2010) or anandamide (Willibald et al. 2012), but as yet this appears to be a largely unexplored area.

Over the years, oligonucleotide chemists have reveled in the “which is the best” arguments: Is an LNA superior to an MOE modification? Is the siRNA better than an ASO? Is a lipid nanoparticle formulation better than a conjugate? This banter extends to the merits of stereopure PS-oligonucleotides (Hyjek-Składanowska et al. 2020; Østergaard et al. 2020), and discussions between those with opposing views will continue, at least until the approval of the first stereopure PS drug settles the question. Based on emerging work, it appears that PS stereochemistry has much to offer in terms of improving the PK and PD properties of oligonucleotides. The challenge here is to design experiments that can link a particular fingerprint of PS stereochemistry to the desired property of interest.

In conclusion, young chemists rest assured: there is still a need for innovation in the oligonucleotide therapeutics field.
